# Climate Change, Human Health, and Health Informatics: A New View of Connected and Sustainable Digital Health

**DOI:** 10.3389/fdgth.2022.869721

**Published:** 2022-03-15

**Authors:** Kathleen Gray

**Affiliations:** Centre for Digital Transformation of Health, University of Melbourne, Melbourne, VIC, Australia

**Keywords:** carbon footprint, climate change, digital health, environmental health, public health

## Abstract

The connection between human health and climate change has had a scientific basis for many decades. However, little attention has been directed to applying the science of health informatics to this aspect of health and healthcare until recently. This paper briefly reviews examples of recent international work on two fronts: to consider how health informatics can reduce the carbon footprint of healthcare, and to consider how it can integrate new kinds of data for insights into the human health impacts of climate change. Health informatics has two principles of fundamental relevance to this work - connectedness, in other words linking and integrating health data from multiple sources; and sustainability, in other words making healthcare overall more efficient and effective. Deepening its commitment to these principles will position health informatics as a discipline and a profession to support and guide technological advances that respond to the world's climate health challenges.

## Introduction: Health Informatics Turns Its Gaze to Climate Change

Climate change is the major human health issue of our time. “Code red for a healthy future” was the subtitle of The Lancet report on health and climate change in 2021 (1). Many health disciplines are recognizing that they have a role in addressing this issue. What is the role of health informatics?

The connection between human health and climate change has had a scientific basis for many decades. The Intergovernmental Panel on Climate Change (IPCC) has been building a picture of these connections for over two decades [e.g., (2)]. Key 21st Century science is summarized in a major 2015 report on planetary health (3). The connections are becoming increasingly clear and concerning, as described in many places—for example (4, 5). As part of the United Nations Climate Change Conference in 2021, in the COP26 Health Program, over 50 nations pledged to transform health systems to be more sustainable, i.e., less carbon-emitting, and to build health systems that are resilient to the direct and indirect impacts on health of climate change (6).

Health informatics is the scientific knowledge and professional practice of technological approaches to working with health data, information and knowledge, and it underpins digital health, e-health, m-health, telehealth, and a variety of other terms for information technology applications in health. Health informatics' fundamental aim to improve health and healthcare is expressed most simply in Friedman's theorem, “persons supported by information technology will be better than the same persons performing the same task unassisted” (7); advancement, connection, empowerment, and transformation are some of the more elaborate terms used to echo this idea. In care settings, health informatics may aim to improve effectiveness, safety, appropriateness, continuity, accessibility, efficiency and sustainability. In research settings, health informatics may aim to improve the accuracy, efficiency and scale of methods for analyzing data and communicating knowledge.

Most health informatics attention is directed to improve practices and services in physically and politically established settings, notably in hospitals and primary care clinics, and in clinical and biomedical research organizations. Thus, related concepts—clinical decision support, electronic records and data warehouses, mobile health and telehealth, etc.—dominate bibliometric analyses of the world literature, for example (8–11). Nevertheless, most human health issues and responses occur outside clinical care and biomedical research institutions - economic, social, cultural and environmental factors are key determinants of health. Improving these aspects of health is the domain of public health, and public health informatics historically has received less attention than clinical and research informatics. More recently, consumer technology trends and the COVID-19 pandemic are drawing more attention to this domain of health informatics [for example, (12–14)].

Given that environmental determinants of health have fallen outside the mainstream of health informatics priorities over a long period, it has been rare to find concerted health informatics attention being directed to the health aspects of climate change. However, we can now see a shift toward this. An October 2021 editorial in the Journal of the American Medical Informatics Association signaled expert recognition of the intersection of climate, health, and informatics (15). In the same month, the European Journal of Public Health reported on a workshop on “The Role of Digital Public Health in the European Climate Pact and Green Deal”—this included case studies of digital health interventions showing significant effects on reducing the impact of climate change and outlined a roadmap for further research (16, 17).

Health informatics' improvement aim must be directed more toward human health aspects of climate change. Approaches are emerging to health informatics contributions to tackle this critical health challenge, and there are major new opportunities for health informatics to address the human health aspects of our troubled planetary life support system. This paper briefly reviews some recent international responses, from two perspectives: how health informatics can reduce the carbon footprint of healthcare, and how it can integrate new kinds of data for insights into the human health impacts of climate change.

## Health Informatics Is Needed to Reduce Healthcare's Carbon Footprint

Healthcare is responsible for between 4 and 5% of global carbon dioxide emissions, the biggest carbon footprint of any service sector. Carbon dioxide emissions embedded in goods and services provided by medical retailers, hospitals, ambulatory, long-term, and preventive healthcare services have been calculated and compared across OECD countries and China and India, for the period since 2000 (18). Major contributors to these emissions are medicines (including inhalers), anesthetic gases, patient and staff transport, heating and cooling of facilities, electricity use, waste management, and food and catering services (19).

Furthermore health information and communication technologies themselves can be part of the problem. Globally digital health may contribute to carbon dioxide emissions through the production and disposal of health wearables and medtech devices, through the energy costs of operating data centers and telecommunication centers, and through the increasingly intensive energy consumption of data processing to support health applications of artificial intelligence and machine learning (20, 21).

Health informatics can play a mitigation role in healthcare services' general carbon-emitting practices in several ways. One is by directing health data science efforts toward this; examples show how it is possible to model the emission consequences of decisions in surgical anesthesia and in intensive care, using industrial life cycle assessment software (22, 23). Another approach is to support the expansion of virtual care, by refining telehealth platforms and tools: a systematic review found 14 studies from around the world claiming that telehealth reduces emissions as compared with in-person care, mainly by reducing travel-related fuel consumption. However, the review notes that the calculations in these studies may not give a full account of the carbon footprint of telehealth (24). A higher level approach to this mitigation role is health informatics work to build the new national health information infrastructure needed to support governments to reduce carbon emissions across their entire hospital networks and health systems, such as the UK Delivering a Net Zero NHS initiative (https://www.england.nhs.uk/greenernhs/) and the US Office of Climate Change and Health Equity (https://www.hhs.gov/ocche/index.html). In work at this level, health terminology standards and healthcare information systems need to incorporate emerging metrics of healthcare sustainability, such as Hensher and McGain (25).

To minimize the specific impacts of digital health itself, there is a role for health informatics to provide guidance on low-carbon health IT infrastructure decisions. This will entail adapting generic technology industry standards such as TCO Certified (https://tcocertified.com) for health IT contexts. For example, hospital IT management strategies should include upgrading to energy-efficient equipment and systems and adopting responsible electronic waste disposal practices (26). Further, health informatics can guide wise use of digital and data-driven health technologies, in ways that minimize wasteful over-servicing. For example, algorithms for analyzing large databases of routinely collected clinical data and patient trajectories can be designed not only to support more accurate diagnostic decisions, but also to reduce the likelihood of overdiagnosis that has arisen from proliferation of new digital screening technologies (27).

## Health Informatics Is Needed to Integrate Climate-Related Human Health Data

Climate change effects on human health arise from increased frequency of extreme weather and natural disasters such as hurricanes and wildfires; from growing spread of water-borne, air-borne, food-borne, and vector-borne pathogens; and from diminished air quality, drinking water supplies and food production. The consequences are increased infectious and chronic disease, physical and mental illness, workforce health and safety issues, across human lifespans around the world—although experiences are spread unevenly (28). Examples from The Lancet annual report (1) illustrate the scale: In 2020 close to 300 billion potential working hours were lost due to extreme heat, with agricultural workers in poorer countries carrying around half of this loss. From 1950–59 to 2010–19 conditions for malaria transmission rose by almost 40% in the most populated highland areas in the poorest countries. Millions of deaths annually would be prevented by less air pollution exposure, healthier living situations and nutrition, and more equitable clean energy access.

Many different clinical and public health informatics innovations are emerging that address specific and localized health effects of climate change. For example, Rodríguez-González et al. (29) describes the role of artificial intelligence in predicting and treating infectious disease outbreaks that are influenced by climate change, particularly in tropical climate zones; and Park et al. (30) explores digital health technologies' abilities to reduce the adverse effects of environmental exposure in chronic airway diseases, based on personal exposure-response modeling.

Even more importantly, overarching health informatics approaches are emerging. For instance, researchers reviewed information about extreme heat events, including the many factors that put people at higher risk and the variety of actions people can take in response, provided for citizens on local government websites of 10 major US cities (31). This review found inconsistencies and gaps that point to the need to standardize information content and formats. The formation of a Global Heat Health Information Network (https://ghhin.org/) promises to deliver such standards in an integrated action plan, although to date there is only brief detail about the information architecture and infrastructure to achieve this.

Many other dimensions of climate and health are less well served by health informatics as yet. A comparative review of four climate health monitoring devices for individuals to monitor their personal air pollution exposure continuously (32) reminds us that climate health informatics must integrate and interpret large volumes of data from diverse, non-standard, non-traditional sources. The management at scale of all sorts of climate health data, and the ability to analyze their complex interactions with other human health data for personalized care, will rely on exposome informatics, a field still in development (33). Further advances managing in human health in the era of climate change will require information systems that capture, process, and communicate combined data on human, animal and plant health, in order to work with complex challenges such as syndemics (34). The One Digital Health framework is an example of how health informatics can begin to work systematically with digital technology potentials in relation to human health, animal health and broader ecosystem health (35).

## Conclusion: Health Informatics Has a Responsibility to Connect Climate and Health

This review has focused on two key aspects of the work to be done. The first is to decarbonise the healthcare sector so that it is not contributing to global warming—noting that part of this work is to ensure that the use of health information technology itself does not exacerbate climate change. The second is to use data to gain insights into the health impacts of climate change and their mechanisms, as well as opportunities to intervene in these mechanisms so as to protect and promote health. As well, there are two other important areas of work which hold many opportunities, untapped so far, for health informatics to make a difference: making healthcare services more resilient in the face of increasingly more frequent and severe health-harming climate change impacts; and enabling public health functions to adopt new monitoring and communication practices to minimize preventable health harms from climate change.

“Physicians and the healthcare system are called to an increased awareness of climate issues and to perpetual adaptation and adoption of technological advances to change the way medicine is practiced in the future” (36). Indeed, health informatics solutions already are supporting professional development about climate and health for other parts of the health workforce, such as in an international telementoring service (37). Beyond support services though, health informatics as a discipline and a profession should be providing greater thought leadership about technological advances that respond to the world's climate health challenges.

Professionalism in health informatics is marked by a principled approach to researching and developing health information systems that are connected (38) and sustainable (39). These two principles—connectedness, in other words linking and integrating health data from multiple sources; and sustainability, in other words making healthcare overall more efficient and effective—resonate when we consider climate change and human health. Both of these health informatics principles need to be elaborated and communicated further, to substantiate informatics contributions to mitigation, adaptation and discovery efforts in this field of health.

To play a role in responding to the climate health crisis, health informatics professionals can build increased awareness of climate issues into the aims and focus of their own work; research institutes can provide forums to stimulate innovative thinking on this topic; professional societies can generate responsible practice standards. Health informatics can show leadership by taking a more comprehensive view of connected and sustainable healthcare, and by applying its ideal of improving healthcare through information technology to the immense reality of climate health effects.

Health informatics professionals must do more in their practice to address the challenges associated with protecting human health in a changing climate; other health professionals working to address climate health challenges must take advantage of the science of informatics to achieve sophisticated impacts at meaningful scale.

## Author Contributions

The author confirms being the sole contributor of this work and has approved it for publication.

## Conflict of Interest

The author declares that the research was conducted in the absence of any commercial or financial relationships that could be construed as a potential conflict of interest.

## Publisher's Note

All claims expressed in this article are solely those of the authors and do not necessarily represent those of their affiliated organizations, or those of the publisher, the editors and the reviewers. Any product that may be evaluated in this article, or claim that may be made by its manufacturer, is not guaranteed or endorsed by the publisher.
